# Comparative Effect of Extraction Techniques on the Phenolic Content and Antioxidant and Antigenotoxic Activities of *Rubus saxatilis* L.

**DOI:** 10.3390/plants15172568

**Published:** 2026-08-24

**Authors:** Aknur D. Orazbay, Assel G. Zhumina, Margarita Yu. Ishmuratova, Gayane A. Atazhanova, Yana Levaya, Serikbai K. Abilev, Olga N. Antosyuk, Anastasia K. Verbitskaya

**Affiliations:** 1 Faculty of Biology and Geography, Karaganda National Research University Named After Academician E.A. Buketov, Karaganda 100028, Kazakhstan; ladyasha@bk.ru (A.D.O.); margarita.ishmur@mail.ru (M.Y.I.); atazhanova@qmu.kz (G.A.A.); ylevaya@qmu.kz (Y.L.); 2 School of Pharmacy, Karaganda Medical University, Gogol Street 40, Karaganda 100012, Kazakhstan; 3 Laboratory of Ecological Genetics, Vavilov Institute of General Genetics, Russian Academy of Sciences, Moscow 117971, Russia; abilev@vigg.ru; 4 Faculty of Biology, Lomonosov Moscow State University, Moscow 119234, Russia; 5 Department of Biodiversity and Bioecology, Ural Federal University Named After the First President of Russia B.N. Yeltsin, Mira Street 19, Yekaterinburg 620002, Russia; antosuk-olga@mail.ru; 6 Laboratory of Introduction of Herbaceous Plants, Botanical Garden of the Ural Branch of the Russian Academy of Sciences, 8 Marta Street 202a, Yekaterinburg 620144, Russia; n.vierbitskaia@mail.ru

**Keywords:** *Rubus saxatilis* extracts, phenolic compounds, HPLC-ESI-MS, DPPH, FRAP, lux-biosensors, genotoxicity

## Abstract

*Rubus saxatilis* L. (*Rosaceae*) is used in traditional medicine in Central Kazakhstan, yet the effect of the extraction method on its phenolic profile and biological activity has not been studied systematically. Three extracts were compared: two prepared by ultrasound-assisted extraction differing only in solvent—water (WE) and 50% ethanol (UAE)—and one by microwave-assisted extraction with 50% ethanol (MAE), allowing the effect of solvent (WE vs. UAE) and of extraction technique (UAE vs. MAE) to be assessed separately. Phenolic composition was determined by HPLC-UV-ESI-MS; antioxidant activity in vitro; cytotoxicity for *Artemia salina*; and antigenotoxic effects using *Escherichia coli* K12 MG1655 lux-biosensors (pKatG::lux, pSoxS::lux, pColD::lux) and *Drosophila melanogaster* models. Four phenolics were identified, with gallic acid dominant in the UAE and MAE extracts and hyperoside predominant in WE, while rutin was not detected in WE. MAE showed the strongest radical-scavenging activity (DPPH, IC_50_ = 7.44 µg/mL), whereas WE had the highest reducing power (FRAP, EC_50_ = 52.97 µg/mL). All extracts were non-toxic for *A. salina* and induced neither oxidative stress nor the SOS response; instead, they attenuated dioxidine-induced SOS signalling by up to 57%, suggesting an antigenotoxic (DNA-protective) effect at the bacterial level. In *Drosophila*, extracts produced moderate extract-dependent genotoxicity: the lowest damage was observed for MAE. The extraction method governs the phenolic profile and biological activity of *R. saxatilis*, with MAE being most favorable.

## 1. Introduction

Plant-derived phenolic compounds are increasingly regarded as a promising basis for the development of functional foods, phytopharmaceuticals, and cosmeceuticals. Polyphenols possess pronounced antioxidant activity, contribute to the regulation of redox homeostasis, and can modulate processes associated with DNA damage and the inflammatory response. Given the rising prevalence of disorders linked to oxidative stress, the search for new natural sources of bioactive compounds remains a relevant task in contemporary phytochemistry [1].

The genus *Rubus* L. (Rosaceae) is characterised by a high content of phenolic acids and flavonoids and has been studied extensively as a source of antioxidant compounds [2,3]. The quantitative distribution of these compounds depends markedly on the extraction conditions, including both solvent composition and the extraction technique applied [4,5,6,7]. Ultrasound-assisted extraction is widely used to improve the recovery of phenolic compounds, and its efficiency is further influenced by the choice of solvent, ranging from water (WE) to hydroethanolic mixtures (UAE); microwave-assisted extraction (MAE) represents an alternative intensified approach with distinct extraction kinetics. However, studies systematically comparing the effect of solvent composition within ultrasound-assisted extraction alongside microwave-assisted extraction, within a single species, remain scarce [8,9].

*Rubus saxatilis* L. (stone bramble) is widely distributed across the natural ecosystems of Central Kazakhstan and has a long history of use in folk medicine. While its morphological features have been described previously [10], and phytochemical data are available for certain populations and related taxa, a comprehensive characterization of its phenolic composition and biological activity is still lacking [11,12]. To the best of our knowledge, no previous study has combined phytochemical profiling, antioxidant evaluation, bacterial biosensor-based genotoxicity/antigenotoxicity assays, and in vivo *Drosophila* models within a single framework for *R. saxatilis* or related *Rubus* species—despite the proven value of such multi-level approaches for other phytochemically rich plants.

Lux-biosensor systems based on *Escherichia coli* allow real-time detection of oxidative-stress and SOS-response induction [13,14,15,16]. Complementing these with *Artemia salina* and *Drosophila melanogaster* models extends the analysis to toxic, genotoxic effects in vivo, enabling a more comprehensive biological interpretation.

The aim of the present study was to comparatively assess the influence of extraction solvent (WE vs. UAE) and extraction technique (UAE vs. MAE) on the phenolic profile and the multi-level biological activity of leaf and petiole extracts of *R. saxatilis*.

## 2. Results

### 2.1. Chemical Composition

**Figure 1:**
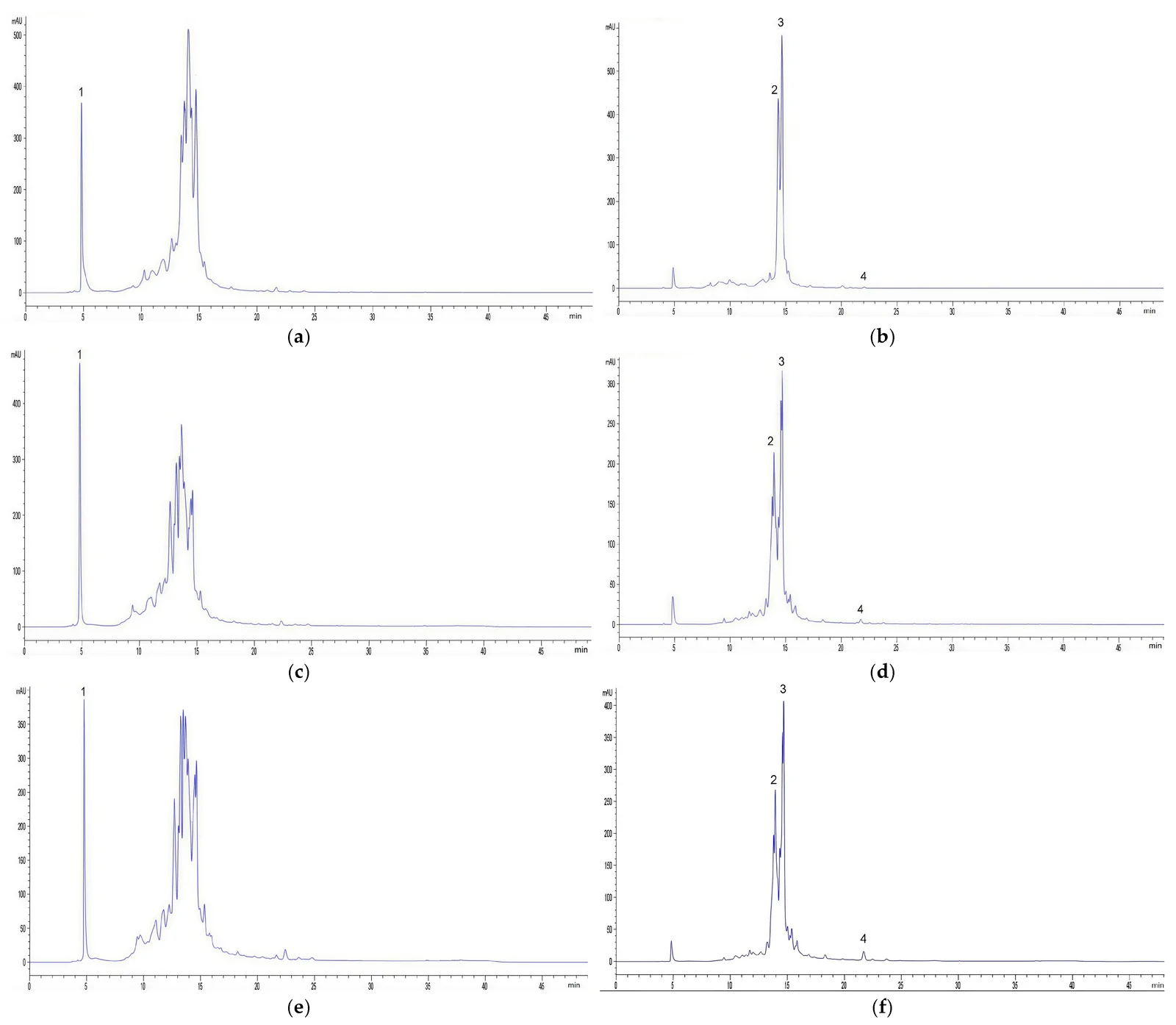
HPLC-UV chromatograms of *R. saxatilis* extracts. (**a**) WE, 280 nm; (**b**) WE, 360 nm; (**c**) UAE extract, 280 nm; (**d**) UAE extract, 360 nm; (**e**) MAE extract, 280 nm; (**f**) MAE extract, 360 nm. Peak numbers correspond to the identified compounds listed in Table 1: 1—gallic acid; 2—rutin; 3—hyperoside; 4—quercetin.

Phytochemical profiling of *R. saxatilis* extracts by HPLC-UV-ESI-MS revealed four polyphenols (Table 1; Figure 1). Gallic acid was the dominant component in UAE and MAE samples 14.68–17.36 mg/g DE, while hyperoside was the most abundant phenolic constituent quantified as 19.75 mg/g of DE in WE extract. Rutin was not detected in WE but was present in UAE (7.28 mg/g of DE) and MAE (8.92 mg/g of DE), reflecting solvent-dependent differences in the recovery of flavonoid glycosides. Quercetin was detected only at low concentrations in all extracts.

### 2.2. Antioxidant Activity

#### 2.2.1. 1,1 Diphenyl-2-picrylhydrazyl Radical (DPPH)-Scavenging Activity

The DPPH assay results are presented in Table 2. The highest radical-scavenging activity was recorded for MAE (IC_50_ = 7.4 µg/mL), whereas the activity of the UAE extract (IC_50_ = 104 µg/mL) and the WE extract (IC_50_ = 232 µg/mL) was 14- and 31-fold lower, respectively, than that of MAE.

#### 2.2.2. Ferric Reducing Antioxidant Power (FRAP)

In the FRAP assay, the highest reducing power was observed for WE (EC_50_ = 52.97 µg/mL), whereas MAE (EC_50_ = 177.49 µg/mL) and UAE (EC_50_ = 237.36 µg/mL) showed moderate activity (Table 3).

### 2.3. Brine Shrimp Lethality Assay

None of the three *R. saxatilis* extracts exhibited cytotoxic activity against *Artemia salina* nauplii: the LC_50_ values exceeded 1000 µg/mL for all samples tested (Table 4). According to the toxicity criteria of Meyer and Clarkson, the extracts are therefore classified as non-toxic.

### 2.4. Lux-Biosensor Assay

The potential pro-oxidant and genotoxic properties of *Rubus saxatilis* extracts were evaluated using a panel of three bioluminescent reporter strains of *Escherichia coli* K12 MG1655: pSoxS::lux (SoxRS regulon, responsive to superoxide anion), pKatG::lux (OxyR regulon, responsive to H_2_O_2_), and pColD::lux (SOS regulon, responsive to DNA damage). Bioluminescence intensity, expressed in relative light units (RLU), was compared with the control (H_2_O) by one-way ANOVA followed by Dunnett’s post hoc test (α = 0.05).

**Figure 2:**
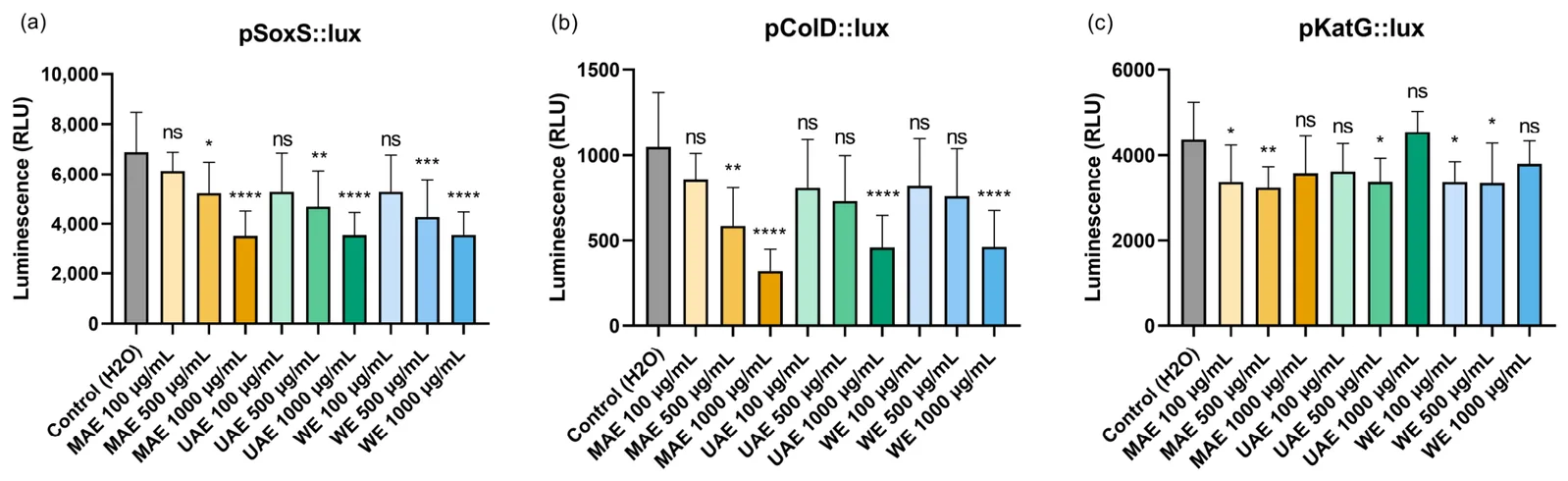
Effects of *Rubus saxatilis* extracts on basal bioluminescence of *Escherichia coli* K12 MG1655 lux-biosensor strains. Luminescence (RLU) of strains carrying the *pSoxS::lux* (**a**), *pColD::lux* (**b**), and *pKatG::lux* (**c**) reporter constructs following 90-min exposure to WE, UAE, and MAE at concentrations of 100, 500, and 1000 µg/mL. H_2_O served as the negative control. Data are presented as mean ± SD (*n* = 9, 8, and 10 for pSoxS::lux, pColD::lux, and pKatG::lux, respectively). Statistically significant differences versus control were determined by one-way ANOVA followed by Dunnett’s post-hoc test (* *p* < 0.05; ** *p* < 0.01; *** *p* < 0.001; **** *p* < 0.0001; ns—not significant).

All three extracts produced a concentration-dependent decrease in basal luminescence of the MG1655 (pSoxS::lux) strain relative to the control (6881 ± 1587 RLU; Figure 2a). ANOVA revealed highly significant between-group differences (F(9, 80) = 7.284; *p* < 0.0001). At 100 µg/mL, none of the extracts caused a statistically significant decrease (MAE: −10.8%, *p* = 0.769; UAE: −23.3%, *p* = 0.061; WE: −23.3%, *p* = 0.060). At 500 µg/mL, significant suppression of luminescence was recorded for MAE (5232 RLU; −24.0%; 95% CI: 2.8–3295.2; *p* = 0.049), UAE (4685 RLU; −31.9%; 95% CI: 549.8–3842.2; *p* = 0.004), and WE (4281 RLU; −37.8%; 95% CI: 953.6–4246.4; *p* < 0.001). At the highest concentration, 1000 µg/mL, all three extracts produced highly significant suppression of luminescence by 48–49% relative to the control: MAE (3525 RLU; *p* < 0.0001), UAE (3550 RLU; *p* < 0.0001), and WE (3561 RLU; *p* < 0.0001); no significant differences between extraction methods were detected at this concentration (*n* = 9 per group).

The MG1655 (pKatG::lux) strain, whose luminescence is regulated by the OxyR-dependent promoter of the katG gene, exhibited a non-monotonic response to the extracts (Figure 2b). Mean basal luminescence in the control was 4363 ± 872.1 RLU. ANOVA revealed significant between-group differences (F(9, 90) = 4.195; *p* = 0.0002). At 100 µg/mL, a significant decrease was recorded for MAE (3370 RLU; −22.8%; 95% CI: 138.2–1847.8; *p* = 0.015) and WE (3368 RLU; −22.8%; 95% CI: 140.1–1849.7; *p* = 0.015), whereas UAE did not reach statistical significance at this concentration (3629 RLU; −16.8%; *p* = 0.126). At 500 µg/mL, significant suppression of luminescence was recorded for all three extracts: MAE (3236 RLU; −25.8%; 95% CI: 272.0–1982.0; *p* = 0.004), UAE (3371 RLU; −22.7%; 95% CI: 137.3–1846.9; *p* = 0.015), and WE (3348 RLU; −23.3%; 95% CI: 160.2–1869.8; *p* = 0.012). At the highest concentration, 1000 µg/mL, none of the extracts showed a significant effect (*p* > 0.05): MAE (3586 RLU; *p* = 0.092) and WE (3803 RLU; *p* = 0.371). Notably, at 1000 µg/mL UAE showed a mean luminescence of 4546 RLU, marginally exceeding the control level (+4.2%; 95% CI: −1038 to 671.9; *p* = 0.996) (*n* = 10 per group).

The MG1655 (pColD::lux) strain, reflecting the SOS-regulated response to DNA damage, showed the most pronounced concentration-dependent decrease in basal luminescence among the three biosensors tested (Figure 2c). Mean control luminescence was 1048 ± 315.9 RLU. ANOVA revealed highly significant between-group differences (F(9, 70) = 6.908; *p* < 0.0001). At 100 µg/mL, the decrease in luminescence was 18–23% for all extracts, but the differences did not reach statistical significance (MAE: *p* = 0.509; UAE: *p* = 0.264; WE: *p* = 0.310). At 500 µg/mL, significant suppression was recorded only for MAE (585.4 RLU; −44.1%; 95% CI: 132.1–793.4; *p* = 0.002), whereas UAE (731.1 RLU; −30.2%; *p* = 0.066) and WE (759.8 RLU; −27.5%; *p* = 0.115) did not reach statistical significance. At 1000 µg/mL, all three extracts produced highly significant suppression of luminescence: MAE (317.9 RLU; −69.7%; 95% CI: 399.6–1061.0; *p* < 0.0001), UAE (459.6 RLU; −56.1%; 95% CI: 257.8–919.2; *p* < 0.0001), and WE (463.5 RLU; −55.8%; 95% CI: 254.0–915.3; *p* < 0.0001) (*n* = 8 per group).

Taken together, the lux-biosensor data showed that none of the *R. saxatilis* extracts, at any of the concentrations tested, caused a significant increase in basal bioluminescence of the reporter strains relative to the control. On the contrary, all three extracts concentration-dependently suppressed the bioluminescence of the pSoxS::lux and pColD::lux strains, reaching a maximal effect at 1000 µg/mL (a 48–70% reduction). The pKatG::lux strain showed the greatest sensitivity at 500 µg/mL, with partial attenuation of the effect at 1000 µg/mL.

#### 2.4.1. Lux-Biosensor Assays with Inducers of Oxidative Stress and the SOS Response

To assess the ability of *R. saxatilis* extracts to modulate induced stress responses, the extracts were co-applied with the corresponding inducers: dioxidine (0.0005 mmol/mL) for the MG1655 (pSoxS::lux) and MG1655 (pColD::lux) strains, and hydrogen peroxide (0.03%) for the MG1655 (pKatG::lux) strain. Luminescence induced by the inducer alone, without extract, served as the reference.

**Figure 3:**
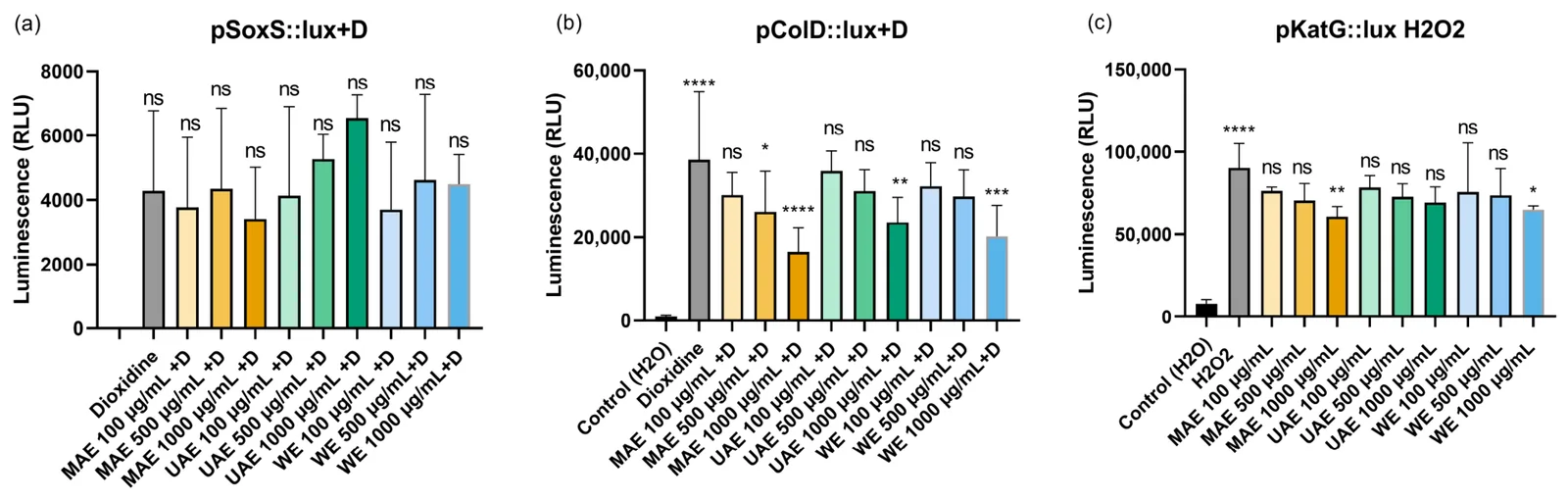
Modulation of bioluminescence in *E. coli* K12 MG1655 lux-biosensor strains by *Rubus saxatilis* extracts under induced stress conditions. Luminescence (RLU) of strains carrying pSoxS::lux (**a**), pColD::lux (**b**), and pKatG::lux (**c**) reporter constructs following co-exposure to WE, UAE, or MAE at 100, 500, and 1000 µg/mL combined with dioxidine (0.0005 mmol/mL; panels (**a**,**b**)) or hydrogen peroxide (0.03%; panel **c**). The respective inducer alone served as the reference control. Data are presented as mean ± SD *(n* = 6, 7, and 5 for pSoxS::lux, pColD::lux, and pKatG::lux, respectively). Statistically significant differences versus the inducer control were determined by one-way ANOVA followed by Dunnett’s post-hoc test (* *p* < 0.05; ** *p* < 0.01; *** *p* < 0.001; **** *p* < 0.0001; ns—not significant).

MG1655 (pSoxS::lux) + dioxidine: One-way ANOVA revealed no statistically significant between-group differences (F(9, 50) = 1.203; *p* = 0.315) (Figure 3a). Mean luminescence in the dioxidine group was 4285 ± 2485 RLU. By Dunnett’s test, none of the extracts at any concentration produced a significant change in luminescence relative to the dioxidine control (*p* = 0.29 to >0.99 in all cases). MAE at all concentrations (3418–4350 RLU) and WE (3705–4625 RLU) showed a slight downward trend in luminescence; UAE at 500 and 1000 µg/mL, by contrast, tended to increase it (5269 and 6548 RLU, respectively), without reaching statistical significance (*p* = 0.960 and *p* = 0.294) (*n* = 6 per group).

MG1655 (pColD::lux) + dioxidine: Dioxidine produced a marked induction of the SOS response: mean luminescence in the dioxidine group was 38,576 ± 16,179 RLU, 40.8-fold higher than the unstimulated control (945 ± 292.3 RLU; mean difference: 37,631 RLU; 95% CI: 26,336–48,926; *p* < 0.0001). ANOVA revealed highly significant between-group differences (F(10, 66) = 13.58; *p* < 0.0001) (Figure 3b). The Brown–Forsythe and Bartlett tests indicated significant heterogeneity of variances between groups (*p* = 0.019 and *p* < 0.0001, respectively); the Dunnett’s test results are therefore interpreted with this limitation in mind. Co-treatment of the extracts with dioxidine produced a concentration-dependent decrease in induced bioluminescence of 5–57% relative to the dioxidine control. At 100 µg/mL, none of the extracts showed a significant effect (MAE: *p* = 0.242; UAE: *p* = 0.993; WE: *p* = 0.561). At 500 µg/mL, a significant decrease was recorded only for MAE (26,192 RLU; −32.1%; 95% CI: 1089–23,679; *p* = 0.025); UAE (*p* = 0.365) and WE (*p* = 0.204) did not reach statistical significance. At the highest concentration, 1000 µg/mL, all three extracts produced statistically significant suppression of induced luminescence: MAE (16,573 RLU; −57.0%; 95% CI: 10,708–33,298; *p* < 0.0001), UAE (23,625 RLU; −38.7%; 95% CI: 3656–26,246; *p* = 0.004), and WE (20,222 RLU; −47.6%; 95% CI: 7059–29,649; *p* = 0.0002) (*n* = 7 per group).

MG1655 (pKatG::lux) + hydrogen peroxide: Hydrogen peroxide (0.03%) produced a marked induction of OxyR-dependent luminescence: the mean in the peroxide group was 90,312 ± 14,747 RLU, 11.3-fold higher than the unstimulated control (8013 ± 2538 RLU; mean difference: 82,299 RLU; 95% CI: 59,887–104,710; *p* < 0.0001). ANOVA revealed highly significant between-group differences (F(10, 44) = 14.26; *p* < 0.0001) (Figure 3c). The Bartlett test indicated significant heterogeneity of variances (*p* < 0.0001); the results are interpreted with this limitation in mind. Co-treatment with the extracts decreased induced luminescence by 13–33% relative to the hydrogen peroxide group. By Dunnett’s test, a significant decrease was recorded only at the highest concentration: MAE 1000 µg/mL (60,988 RLU; −32.5%; 95% CI: 6913–51,736; *p* = 0.005) and WE 1000 µg/mL (65,220 RLU; −27.8%; 95% CI: 2680–47,504; *p* = 0.021). The remaining groups did not reach statistical significance (*p* = 0.079–0.612) (*n* = 5 per group).

#### 2.4.2. Bacterial Survival Assay

**Figure 4:**
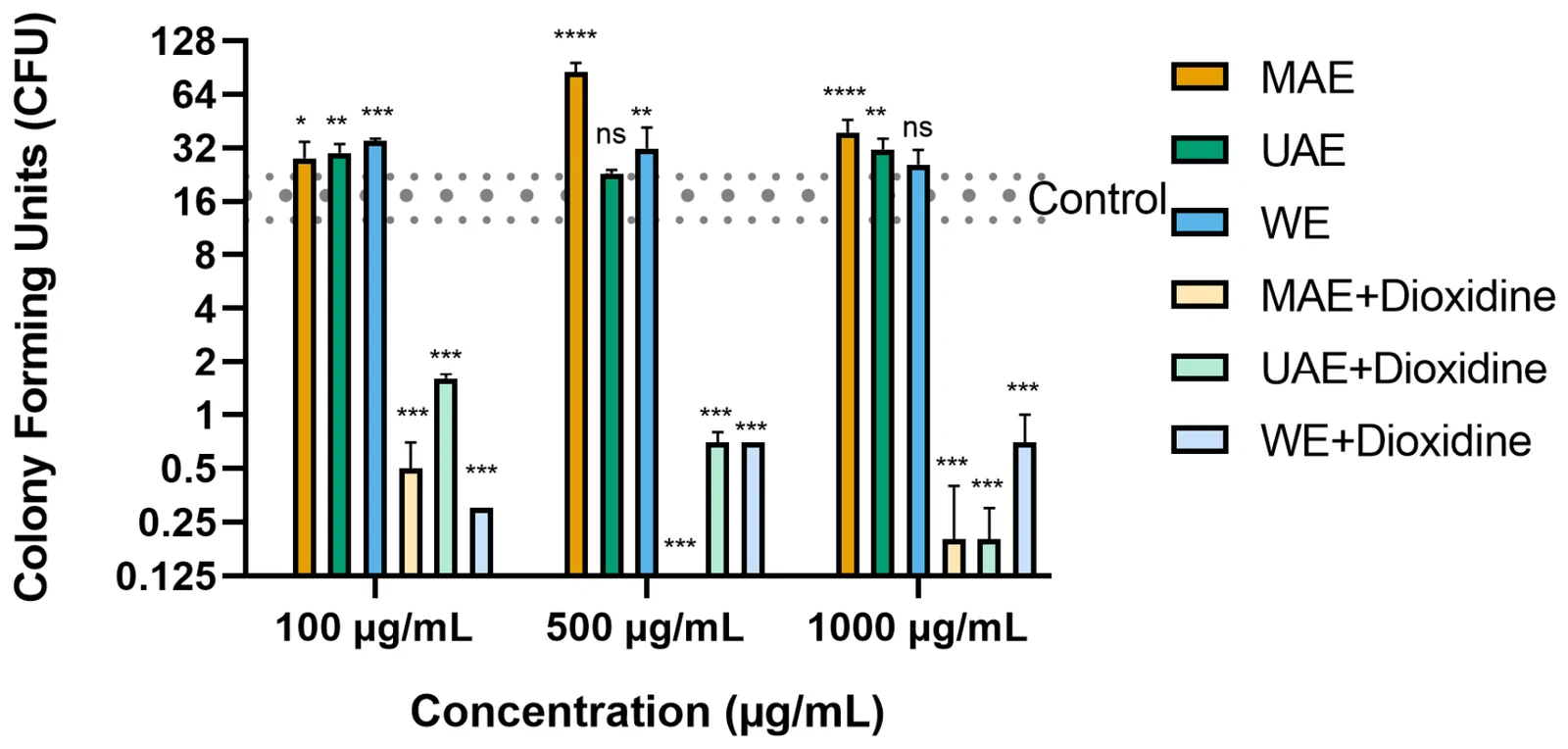
Survival (colony-forming units, CFU) of *E. coli* K12 MG1655 (pColD::lux) after 90-min exposure to *R. saxatilis* extracts (MAE, UAE, WE) alone and in combination with dioxidine (0.0005 mmol/mL) at 100, 500, and 1000 µg/mL. The dotted horizontal band indicates the untreated H_2_O control (mean ± SD = 17.3 ± 4.7 CFU, *n* = 3). In the absence of dioxidine, all three extracts maintained or increased bacterial survival relative to the control (CFU range 23.0 ± 1.0 to 85.3 ± 11.1), indicating the absence of cytotoxic activity. Co-treatment with dioxidine markedly reduced survival (CFU ≤ 1.6), consistent with its antibacterial effect. Data are presented as mean ± SD (*n* = 3). Statistically significant differences versus the inducer control were determined by one-way ANOVA followed by Dunnett’s post-hoc test (* *p* < 0.05; ** *p* < 0.01; *** *p* < 0.001; **** *p* < 0.0001; ns, not significant).

Bacterial survival of *E. coli* K12 MG1655 (pColD::lux) was assessed following 90-min exposure to WE, UAE, and MAE at concentrations of 100, 500, and 1000 µg/mL in the presence or absence of dioxidine (0.0005 mmol/mL). Two-way ANOVA revealed significant effects of extract type (F(6, 56) = 187.7; *p* < 0.0001), concentration (F(3, 56) = 79.60; *p* < 0.0001), and their interaction (F(18, 56) = 49.48; *p* < 0.0001) on CFU counts. All three extracts significantly increased bacterial survival relative to the H_2_O control at all tested concentrations (Dunnett’s test, all *p* < 0.0001), with CFU values ranging from 25.7 ± 5.5% (WE, 1000 µg/mL) to 85.3 ± 11.1% (MAE, 500 µg/mL) (Figure 4). MAE exhibited a non-monotonic concentration–response relationship, with maximal survival at 500 µg/mL (85.3 ± 11.1%) followed by a decline at 1000 µg/mL (39.0 ± 7.0%). WE demonstrated an inverse concentration-dependent pattern, with survival decreasing from 35.0 ± 1.0% at 100 µg/mL to 25.7 ± 5.5% at 1000 µg/mL. UAE showed no significant concentration-dependent trend (30.0 ± 3.6% to 31.3 ± 4.7%). In the presence of dioxidine, no significant differences in CFU counts were observed between extract-treated and control groups at any concentration tested (all *p* > 0.99), indicating absence of antagonistic or synergistic interactions between the extracts and the antibiotic under the conditions studied. The increase in CFU counts indicates that the suppression of bioluminescence is not due to cytotoxicity; it is therefore consistent with, though not direct proof of, an intracellular antioxidant effect of *R. saxatilis* extracts.

#### 2.4.3. Culture Growth Rate Assay

**Figure 5:**
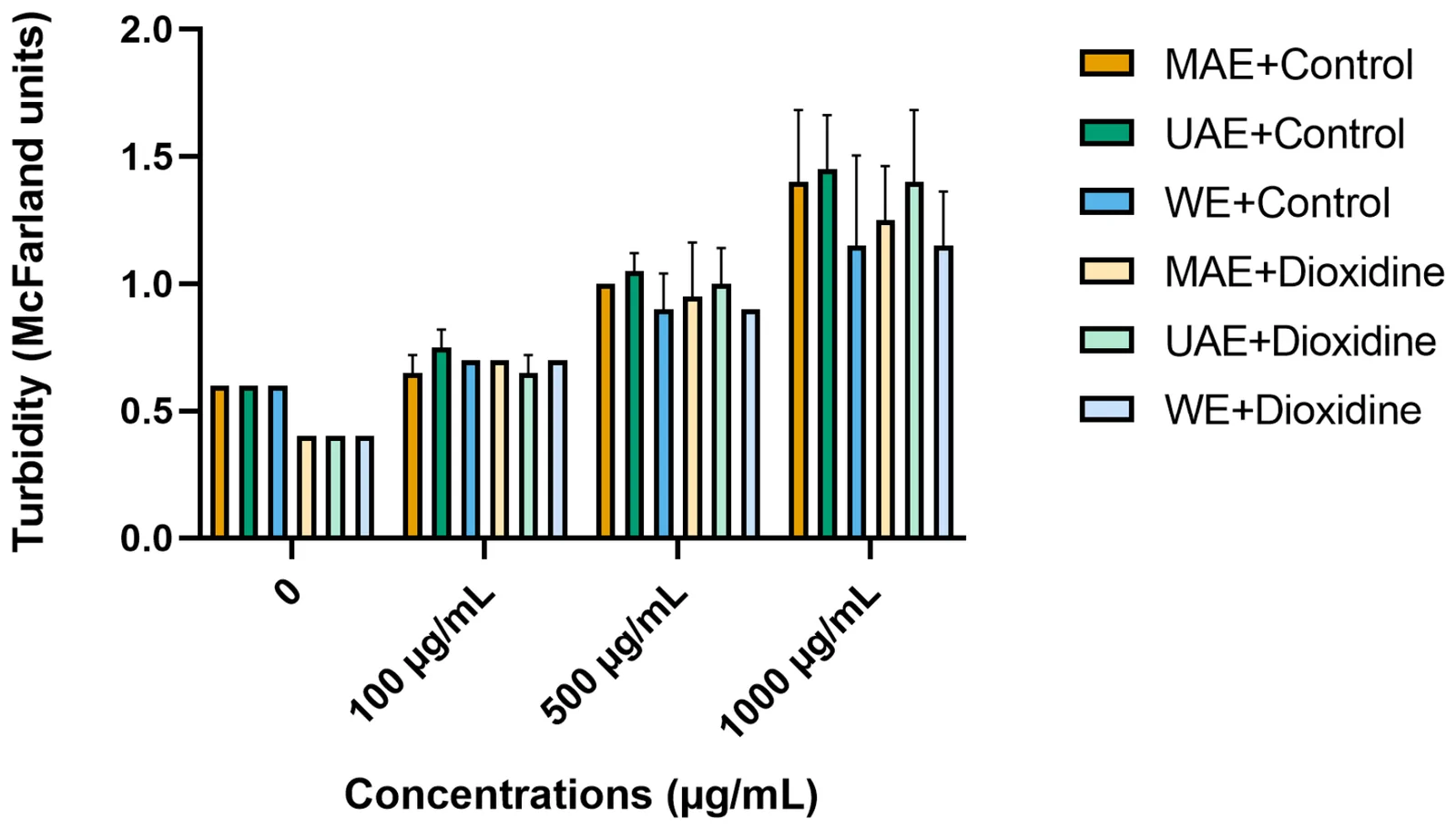
Initial turbidity (McFarland units) of *E. coli* K12 MG1655 (pColD::lux) culture at the start of the experiment following addition of *R. saxatilis* extracts (MAE, UAE, WE) at concentrations of 0, 100, 500, and 1000 µg/mL in the absence (+Control) and presence (+Dioxidine) of dioxidine (0.0005 mmol/mL). A significant effect of concentration on initial turbidity was detected (two-way ANOVA: F(3, 48) = 99.52; *p* < 0.0001), while the effect of extract type (*p* = 0.069) and their interaction (*p* = 0.602) were not statistically significant. Data are presented as mean ± SD (*n* = 3).

Preliminary analysis revealed that all *R. saxatilis* extracts exhibited intrinsic turbidity that increased proportionally with concentration. Two-way ANOVA demonstrated a significant effect of concentration on initial turbidity (F(3, 48) = 99.52; *p* < 0.0001), while the effect of extract type (F(5, 48) = 2.209; *p* = 0.069) and the concentration × extract interaction (F(15, 48) = 0.868; *p* = 0.602) were not statistically significant, indicating that initial turbidity was independent of extraction method. Accordingly, all subsequent growth dynamics are expressed as turbidity change (ΔE = E_t − E_0_) (Figure 5).

**Figure 6:**
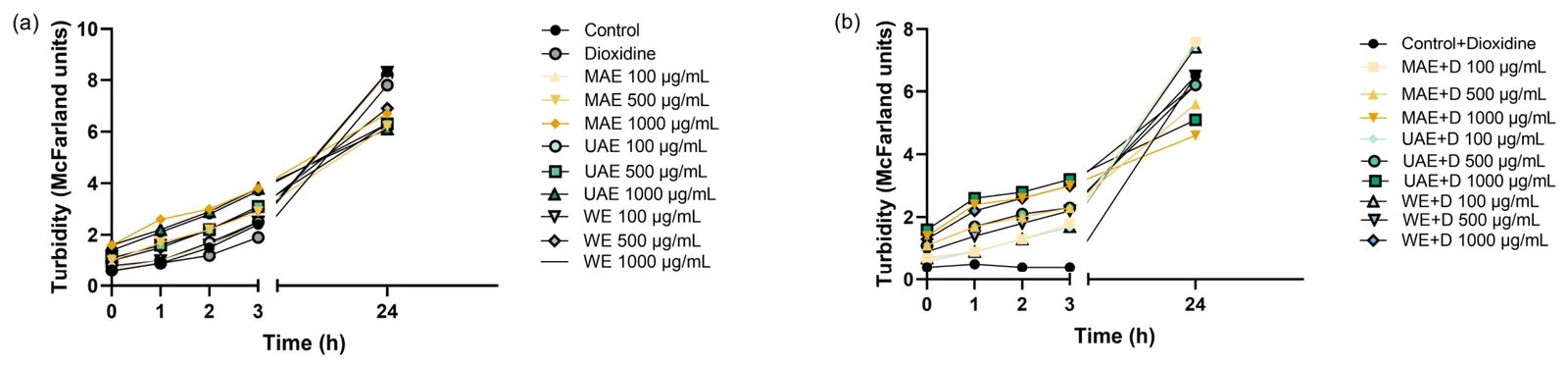
Changes in culture turbidity (ΔE, McFarland units) of *E. coli* K12 MG1655 (pColD::lux) over time following exposure to *R. saxatilis* extracts (MAE, UAE, WE) at concentrations of 100, 500, and 1000 µg/mL in the absence (**a**) and presence (**b**) of dioxidine (0.0005 mmol/mL). No statistically significant differences versus the corresponding control were detected at any time point or concentration tested (one-way ANOVA followed by Dunnett’s post-hoc test; all *p* > 0.05). Data are presented as mean ± SD (*n* = 3).

Turbidity increased over time in all experimental groups. In the absence of dioxidine, one-way ANOVA revealed no statistically significant differences in ΔE between any extract-treated group and the H_2_O control across the entire observation period (F(10, 44) = 0.071; *p* > 0.999; Dunnett’s test, all *p* > 0.99), indicating that none of the extracts significantly altered bacterial growth kinetics under standard conditions (Figure 6a). In the presence of dioxidine, bacterial growth continued in all groups over the 24 h observation period; however, no statistically significant differences in ΔE were detected between extract-treated groups and the dioxidine-only control at any concentration tested (F(9, 40) = 0.162; *p* = 0.997; Dunnett’s test, all *p* > 0.90; Figure 6b). The dioxidine-only group reached 52.1% of the H_2_O control turbidity at 3 h but recovered to control levels by 24 h.

### 2.5. In Vivo Genotoxic Effects on Drosophila melanogaster Meig.

In the control group, cells with intact DNA predominated (Type 0, 72.7%), Type I (minimal) damage accounted for 25.5%, and a small proportion of Type II damage (1.8%) was observed; no Type III or Type IV comets were detected.

**Figure 7:**
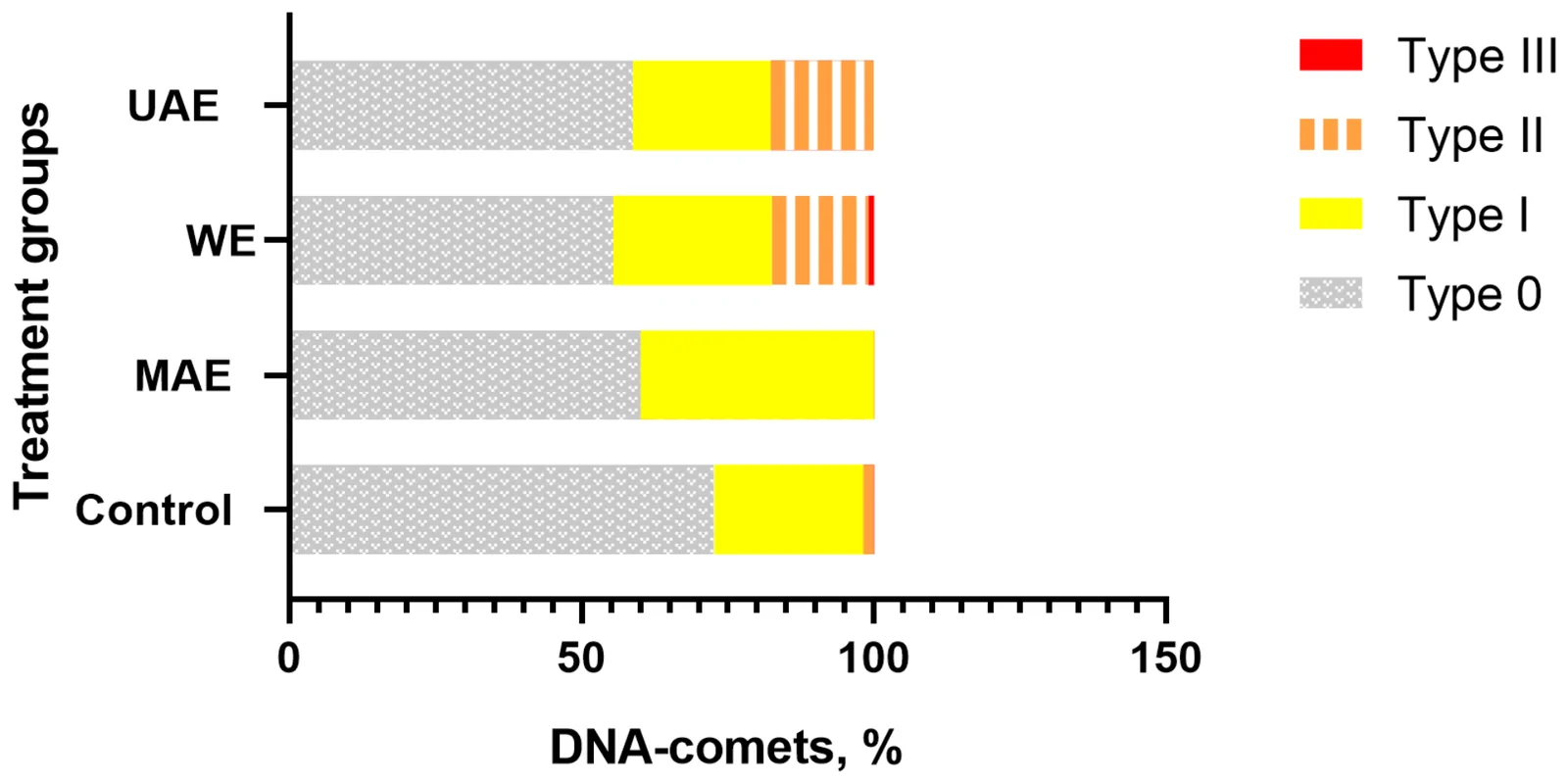
Genotoxic effects of *R. saxatilis* extracts assessed in *Drosophila melanogaster Meig*.

In all extract-treated groups, the proportion of cells with intact DNA (Type 0) was lower than in the control, indicating a genotoxic effect. The least pronounced effect was recorded for MAE, where only Type 0 (60%) and Type I (40%) comets were detected, with no more severe forms of DNA fragmentation. In the WE group damage extended up to Type III (Type 0, 55.3%; Type I, 27.2%; Type II, 16.5%; Type III, 1.0%), whereas in the UAE group the most severe class observed was Type II (Type 0, 58.8%; Type I, 23.5%; Type II, 17.6%), indicating a more pronounced genotoxic potential of these extracts compared with MAE. No cells with maximal DNA fragmentation (Type IV) were detected in any group. The proportion of cells with intact DNA in the WE and UAE groups was comparable (~57%) and substantially lower than in the control (Figure 7).

## 3. Discussion

### 3.1. Phenolic Profile of the R. saxatilis Extracts

HPLC-UV-ESI-MS profiling identified four quantified phenolic compounds: gallic acid, rutin, hyperoside, and quercetin. Although the qualitative phenolic profiles were similar among the extracts, marked quantitative differences were observed. These variations depended not only on the extraction technique but also on the extraction solvent, whose polarity strongly influences the solubility and recovery of individual phenolic compounds. Such solvent-dependent differences in phenolic composition have been widely reported for *Rubus* species [17].

Gallic acid predominated in the UAE (17.36 mg/g DE) and MAE (14.68 mg/g DE), whereas hyperoside was the major quantified phenolic constituent in the WE (19.75 mg/g DE). The higher gallic acid content in the hydroethanolic extracts suggests that aqueous ethanol provided more favorable conditions for the extraction of low-molecular-weight phenolic acids than water alone. Similar observations have been reported for *R. matsumuranus* leaves, in which gallic acid was quantified at 2.03 mg/g dry extract (DE) [17,18,19,20].

Rutin was not detected in the WE but was quantified in the UAE (7.28 mg/g DE) and MAE (8.92 mg/g DE), indicating that its recovery depended primarily on the use of aqueous ethanol as the extraction solvent. The improved extraction of rutin may also be attributed to enhanced solvent penetration and cell-wall disruption promoted by ultrasonic cavitation and microwave irradiation [21,22].

Conversely, hyperoside reached its highest concentration in the water extract (19.75 mg/g DE) and decreased markedly in the hydroethanolic extracts, suggesting a pronounced solvent-dependent distribution of this flavonoid glycoside. Similar differences in hyperoside recovery associated with solvent composition have been reported for *R. idaeus* and other *Rubus* species [23,24,25,26].

This predominance is consistent with earlier reports on *R. saxatilis*, in which hyperoside was structurally confirmed by NMR as hyperin (quercetin 3-*O*-β-D-galactoside) and identified as the major flavonoid glycoside of the species’ leaves [11,12]. These leaves also showed the highest flavonol content among the wild *Rubus* taxa examined (0.56% aglycones after hydrolysis, with a hyperoside level of ≈0.92%, or 9.2 mg/g) [27,28]—in line with the predominance of hyperoside and the low free-quercetin levels found in the present extracts. Direct quantitative comparison with these earlier reports is limited by both analytical and solvent-related differences: the earlier studies applied acidic methanolic hydrolysis to release flavonoid aglycones (quercetin, kaempferol) prior to colorimetric or HPLC quantification, whereas the present study determined intact glycosides without hydrolysis using pure water (WE) or 50% ethanol (UAE, MAE) under ultrasound- and microwave-assisted conditions. This methodological distinction likely accounts, at least in part, for the divergence between the previously reported aglycone-based profile and the glycoside-based profile identified here. Quercetin was detected only at trace concentrations (0.08–0.27 mg/g DE), with the highest level in the MAE extract, possibly reflecting the greater efficiency of microwave-assisted extraction in releasing less polar aglycones. In contrast, gallic acid and rutin—two of the main constituents quantified here, particularly in the hydroethanolic extracts—were not among the phenolics determined in these earlier studies [11,12]; their detection therefore extends the known phenolic profile of the species.

### 3.2. Correlation Between Phenolic Profile and In Vitro Antioxidant Activity

Differences in antioxidant activity reflected the distinct mechanisms assessed by the DPPH and FRAP assays. Among the investigated extracts, the MAE extract exhibited the strongest DPPH radical-scavenging activity, with an IC_50_ below the lowest tested concentration (50 µg/mL), indicating higher radical-scavenging capacity than the WE and UAE extracts. In contrast, the WE extract demonstrated the highest ferric reducing antioxidant power (FRAP; EC_50_ = 52.97 µg/mL). These assays evaluate different antioxidant mechanisms: DPPH primarily reflects hydrogen atom transfer and free radical scavenging, whereas FRAP measures single-electron transfer through the reduction of Fe^3+^ ions. The superior DPPH activity of the MAE extract is consistent with its relatively higher contents of rutin (8.92 mg/g DE), hyperoside (8.70 mg/g DE), and quercetin (0.27 mg/g DE), whereas the stronger FRAP activity of the WE extract may be associated with its higher hyperoside content (19.75 mg/g DE). Previous studies have demonstrated that hyperoside possesses strong antioxidant activity, which is largely attributed to its structural features, including the 3′,4′-dihydroxyl groups on the B ring that facilitate electron donation and free radical scavenging [19]. These findings indicate that the antioxidant properties of *R. saxatilis* extracts are determined by both the extraction solvent and extraction technique, which together govern the recovery of individual phenolic compounds and their relative abundance in the final extracts [18].

### 3.3. Lux-Biosensor Assays: Absence of Pro-Oxidant Effects and Redox-Modulatory Activity

None of the extracts induced significant activation of the oxyRS, soxRS, or SOS regulons, providing no evidence of pro-oxidant activity or direct DNA damage under bacterial exposure. Instead, all three produced dose-dependent suppression of basal bioluminescence, most pronounced for pSoxS::lux (−48–49%) and pColD::lux (−56–70%) at 1000 µg/mL, indicating reduced constitutive superoxide and basal SOS signalling—consistent with an intracellular antioxidant effect rather than cytotoxicity, as confirmed by increased CFU counts.

The pKatG::lux non-monotonic response (significant at 500 µg/mL, attenuated at 1000 µg/mL, UAE +4.2% at 1000 µg/mL) may reflect biphasic redox modulation: moderate concentrations preferentially scavenge H_2_O_2_, while higher concentrations introduce competing effects on sensor kinetics or membrane permeability. Similar hormetic patterns are described for plant polyphenols in prokaryotic redox reporters [27,28,29,30].

### 3.4. DNA-Protective Effects Versus Comet-Assay Genotoxicity: Possible Mechanistic Interpretation

Co-exposure of pColD::lux with dioxidine produced dose-dependent attenuation of SOS-regulon induction (at 1000 µg/mL: MAE −57.0%, UAE −38.7%, WE −47.6%; all *p* ≤ 0.0004); MAE and WE also attenuated H_2_O_2_-induced OxyR luminescence (−32.5% and −27.8%; *p* < 0.01). These suggest that *R. saxatilis* extracts attenuate bacterially induced genotoxic signalling. ROS scavenging, iron chelation, and reduction in Fenton-type lesions are possible underlying mechanisms, but these were not tested here and remain hypothetical.

The apparent discrepancy between the DNA-protective lux signal and increased DNA damage in the *Drosophila* comet assay may be explained by several non-mutually exclusive hypotheses, none of which was tested experimentally in the present study. First, the assays measure distinct endpoints in phylogenetically distant organisms: lux-biosensors detect prokaryotic stress-regulon transcription over 90 min, whereas the comet assay measures strand-break accumulation in eukaryotic cells after dietary exposure over a developmental period—precluding direct comparison. Second, polyphenols undergo cytochrome P450-mediated biotransformation in eukaryotes to electrophilic quinone metabolites capable of forming DNA adducts, a pathway absent in the prokaryotic system; this may explain why compounds that suppress bacterial SOS signalling can simultaneously generate eukaryotic strand breaks. Third, at the highest concentrations, pro-oxidant behaviour may emerge from auto-oxidation of polyhydroxylated polyphenols or transition-metal-mediated Fenton reactions—a concentration-dependent antioxidant-to-pro-oxidant switch documented for gallic acid and flavonol glycosides at elevated doses [31,32,33,34]. Alternatively, the reduced lux signal may partly reflect non-specific effects on the reporter system itself—such as optical interference from extract colour and turbidity, or changes in luciferase kinetics and membrane permeability—and general shifts in cellular metabolism, rather than a specific antioxidant or DNA-protective action.

Importantly, DNA comet assay damage remained well below the etoposide positive control, and MAE—the extract with the highest phenolic complexity and DPPH activity—showed the least genotoxic potential (Type II/III absent). This supports the interpretation that the observed strand breaks reflect moderate, concentration-dependent genotoxicity rather than a primary genotoxic mechanism within the concentration range tested.

### 3.5. Comparison with Other Rubus Species

The phenolic profile of *R. saxatilis* is consistent with the genus-level dominance of gallic acid and flavonol glycosides, mirroring *R. fruticosus* and *R. idaeus*, where UAE and MAE enhance flavonoid glycoside extraction—reflected here by higher rutin in UAE and MAE versus WE. Unlike several studies where *Rubus* polyphenols showed pro-oxidant effects in cellular models, no pro-oxidant activation of bacterial regulons was detected here, possibly reflecting the specific composition of *R. saxatilis* or the concentration range used. This study extends comparative *Rubus* research by integrating lux-biosensors with in vivo *Drosophila* models, not previously reported for this species.

#### Study Limitations

Several methodological and interpretive limitations should be taken into account when evaluating the present findings. First, all extracts were obtained from a single pooled batch of plant material collected at one site; consequently, the replicates reported here represent analytical (technical) rather than independent biological replicates, which constrains the strength of the statistical comparisons drawn between extraction protocols. Second, solvent and extraction technique were not varied fully independently: only the comparison between UAE and MAE isolates the effect of the extraction technique at a constant solvent, whereas the WE–UAE comparison reflects a difference in solvent composition; accordingly, causal statements attributing compositional or activity differences specifically to the extraction technique should be made with caution. Third, the residual moisture content of the raw material was not determined, and the HPLC quantification was performed without a full analytical validation, as the limits of detection and quantification, calibration ranges and coefficients of determination, recovery, and intra- and inter-day precision were not established; the compound contents in Table 1 are therefore reported as single values without dispersion estimates and should be regarded as indicative rather than definitively validated. Fourth, in the DPPH assay the IC_50_ of the MAE extract fell below the lowest tested concentration (50 µg/mL) and was thus obtained by extrapolation, while the FRAP results, expressed as EC_50_, were not referenced to an Fe(II) calibration curve; these values should be interpreted with corresponding caution.

Additional limitations concern the biological models employed. The lux-biosensor response is an indirect, optical read-out that may be influenced by the intrinsic colour and turbidity of the extracts and by non-specific effects on luciferase kinetics, membrane permeability, or general bacterial metabolism; the observed reduction in luminescence is therefore consistent with, but does not by itself constitute direct proof of, an intracellular antioxidant or DNA-protective effect. The mechanisms proposed to reconcile the bacterial and Drosophila data—including reactive oxygen species scavenging, iron chelation, cytochrome P450-mediated formation of quinone metabolites and DNA adducts, Fenton-type reactions, and hormesis—were not investigated experimentally and are presented as hypotheses that require dedicated validation. The comet-assay results, obtained in a single eukaryotic model, would further benefit from more complete reporting of exposure conditions and replicate structure, and should be confirmed using established, orthogonal assays. Finally, no pharmacokinetic, bioavailability, or tissue-concentration data were generated in the present study; consequently, no conclusions regarding the pharmacological, therapeutic, or phytopharmaceutical relevance of the extracts can be drawn, and such applications remain to be established in future research.

## 4. Materials and Methods

### 4.1. Plant Material

#### 4.1.1. Taxonomic Identification

The study object was *Rubus saxatilis* (stone bramble; family Rosaceae Juss.). Species identity was confirmed by the botanist M.Yu. Ishmuratova; a voucher specimen (No. QAR00364) is deposited in the herbarium of the Faculty of Biology and Geography (acronym QAR) of Karaganda Buketov National Research University (Karaganda, Kazakhstan).

#### 4.1.2. Collection of Plant Material

Leaves with petioles of *R. saxatilis* were collected at the fruiting stage (first ten days of August 2023) in Central Kazakhstan, the Karagash tract, Belodymovsky branch, Buyratau State National Nature Park (51.37461° N, 72.38260° E, 400 m a.s.l.). Leaves with petioles from 150 individual plants were pooled into a single composite batch. The macroscopic and microscopic characterization of the *R. saxatilis* raw material (leaves with petioles) was carried out as part of the pharmacognostic standardization of this species; the detailed anatomical description is reported in our previous work [10]. The raw material was dried indoors, protected from direct sunlight, for five days, with regular turning.

### 4.2. Extraction Procedure

Two extracts were prepared from air-dried *R. saxatilis* raw material (aerial part) by ultrasound-assisted extraction, and one by microwave-assisted extraction (MAE), allowing the effect of solvent to be assessed at constant technique (WE vs. UAE) and the effect of extraction technique at constant solvent (UAE vs. MAE).

Ultrasound-assisted extraction (WE and UAE): Air-dried raw material (10 g; particle size 3–5 mm) was extracted without prior soaking with either 50% aqueous ethanol (UAE) or distilled water (WE), at a raw material-to-solvent ratio of 1:20, in a VGT-1200 ultrasonic bath at an ultrasound frequency of 40 kHz and room temperature (20–22 °C) for 30 min. The extraction was repeated three times. The combined extracts (separately for each solvent) were filtered through a paper filter, and the solvent was removed on a rotary evaporator at 50 °C, with subsequent evaporation of the residual solvent to a thick mass. The water-based ultrasound extract is hereafter designated WE and the ethanol-based one UAE; the two differ only in solvent under identical ultrasound conditions. The yield of dry extract was 1.97 g (UAE, 50% ethanol; 19.7% relative to the mass of the initial raw material) and 2.51 g (WE, water; 25.1% relative to the mass of the initial raw material).

Microwave-assisted extraction (MAE): Microwave-assisted extraction was carried out as follows. Air-dried raw material (20 g) was placed in a 500 mL round-bottom flask, 200 mL of 50% aqueous ethanol was added (raw material-to-solvent ratio 1:10), and the flask was placed in a domestic microwave oven (2.45 GHz) fitted with a reflux condenser to condense the vapours. Extraction was carried out over four cycles until a nearly clear solution was obtained, with a total microwave irradiation time of 4 min at a power of 300 W, applied in 10-s pulses separated by 1-min intervals to prevent overheating and boiling of the solvent. The temperature of the mixture was monitored every minute with an IR-T1 CONDTROL infrared thermometer (CONDTROL, Markt Schwaben, Germany). The extract was concentrated under vacuum on a Labtech IR-1LT rotary evaporator (Beijing LabTech Instruments Co., Ltd., Beijing, China), and the dry extract was obtained by vacuum drying at a temperature not exceeding 40 °C, yielding a stable powder. The yield of dry MAE extract was 0.5159 g.

Air-dried plant raw material was used in the study. The residual moisture content of the samples was not determined, as this parameter was not part of the study design. The dried extracts were stored in hermetically sealed penicillin vials in a refrigerator until further analysis.

### 4.3. HPLC-UV-ESI-MS Analysis

The analysis was conducted on an Agilent 1260 Infinity HPLC system (Agilent Technologies, Santa Clara, CA, USA) equipped with a G1314C 1260 VWD VL+ variable-wavelength UV detector and a G6130A LC/MS quadrupole mass spectrometer (Agilent Technologies, Santa Clara, CA, USA). The following reagents were used in the study: acetonitrile for HPLC (≥99.9%, Sigma-Aldrich, Saint-Quentin-Fallavier, France), formic acid (99–100%, AnalaR NORMAPUR^®^, VWR Chemicals, Fontenay-sous-Bois, France), and highly purified water prepared using a Milli-Q water purification system (Millipore, Molsheim, France). The four selected phenolic compounds, standards (gallic acid monohydrate (≥99% HPLC), rutin hydrate (≥94% HPLC), hyperoside (≥97.0% HPLC), quercetin (≥95% HPLC) were purchased from Sigma–Aldrich (USA). Separation was performed on a “Zorbax Eclipse Plus C18” column (150 × 4.6 mm, 3.5 μm, Agilent Technologies, USA) following the procedure described in [35]. A gradient elution mode was applied using mobile phase A (2.5% formic acid in water, *v*/*v*) and mobile phase B (2.5% formic acid in acetonitrile, *v*/*v*). The gradient program was as follows: 0.00 min—3% B; 7.00 min—20% B; 7.10 min—30% B; 27.00 min—40% B; 35.00 min—50% B; 35.10 min—20% B; 40.00 min—3% B. The flow rate was maintained at 0.4 mL/min, and the column temperature at 30 °C. The extracts and standards were dissolved in a mixture of solvents acetonitrile: water = 1:1 (*v*/*v*). The injection volume was 20 µL for the extracts and standards. UV detection wavelengths were 280 nm and 360 nm. Mass spectrometric detection on the G6130A LC/MS system was carried out in negative ionization mode with the following optimized settings: capillary temperature 350 °C, nitrogen drying gas at 8 L/min, and nebulizer pressure 45 psi. Data acquisition and processing were performed using ChemStation B.0403 SP1 software (Agilent Technologies, USA). Identification of analytes was based on comparison of the obtained mass spectra with reference standards and published spectral data. The quantitative content of phenolic compounds in the ultrasonic extract was calculated by the method of an external standard.

### 4.4. Determination of Antioxidant Activity

The antioxidant activity of the extracts was evaluated by two spectrophotometric methods—DPPH and FRAP—based on different mechanisms of antioxidant action.

#### 4.4.1. DPPH (2,2-Diphenyl-1-picrylhydrazyl) Assay

The method is based on the ability of antioxidants to reduce the stable DPPH radical [36,37]. To 100 µL of sample dissolved in ethanol, 3 mL of a 6 × 10^−5^ M ethanolic DPPH solution (Sigma-Aldrich, USA) was added. The mixture was incubated in the dark at room temperature for 30 min, after which the absorbance was measured at 517 nm on a spectrophotometer (Agilent Technologies, Petaling Jaya, Malaysia). Ascorbic acid was used as the positive control. The following concentrations of the extracts and the positive control were tested: 50, 100, 250, 500, and 1000 µg/mL. The radical inhibition percentage was calculated using the formula:

I (%) = (A_0_ − A_1_)/A_0_ × 100

where A_0_ is the absorbance of the control (DPPH without sample) and A_1_ is the absorbance of the test sample. The IC_50_ value was calculated from the inhibition-versus-concentration curve.

#### 4.4.2. FRAP (Ferric Reducing Antioxidant Power) Assay

The method is based on the ability of antioxidants to reduce Fe^3+^ to Fe^2+^ in the presence of the TPTZ reagent (2,4,6-tripyridyl-s-triazine) [38,39]. To 100 µL of sample dissolved in distilled water, 2.8 mL of working FRAP reagent was added, consisting of acetate buffer (0.3 M, pH 3.6), 10 mM TPTZ in 40 mM HCl, and 20 mM FeCl_3_·6H_2_O in a 10:1:1 ratio. The mixture was incubated at 37 °C for 10 min, after which the optical density was measured at 593 nm on a spectrophotometer (Agilent Technologies, Malaysia). Ascorbic acid at 50, 100, 250, 500, and 1000 µg/mL was used as the positive control. The results were expressed as EC_50_ (µg/mL).

### 4.5. Brine Shrimp Lethality Assay

The method is based on a lethality test using *Artemia salina* larvae as the test organism [40,41]. *Artemia salina* eggs were incubated in artificial seawater under aeration at 25 °C for 48–72 h until hatching. After hatching, 20–40 nauplii in 990 µL of seawater were placed in a 2 mL well of a laboratory plate, after which 10 µL of extract solution at the appropriate concentration was added. The number of dead individuals was counted after 1, 4, and 24 h. The mortality percentage (P) was calculated using the following formula:

P = (A − N) × 100%/Z

where A is the number of dead larvae in the test, N is the number of dead larvae before the start of the experiment, and Z is the total number of larvae in the well at the start of the experiment. The experiment was performed in three independent replicates. The concentrations tested were 100, 250, 500, and 1000 µg/mL. The LC_50_ value was calculated by probit regression.

### 4.6. Lux-Biosensor Assay

The biosensors in this test system are genetically modified strains of *Escherichia coli* K12 MG1655 carrying a hybrid plasmid with a regulatory region and the reporter genes of the *luxCDABE* operon, which encodes luciferase and reductase. The *luxAB* genes encode the luciferase subunits, while the *luxCDE* genes encode the reductase required to form the luciferase substrate (tetradecanal) from fatty acids. Activation of the regulatory region by specific signalling molecules is accompanied by intracellular accumulation of luciferase and reductase, causing the bacteria to luminesce. Luminescence was recorded with a Stat Fax 4400 (LuMate) plate luminometer (Awareness Technology, Inc., Palm City, FL, USA) and expressed in relative light units (RLU) [42].

The biosensors were provided by G.B. Zavilgelsky and I.V. Manukhov (State Research Institute of Genetics and Selection of Industrial Microorganisms, National Research Centre “Kurchatov Institute”, Moscow, Russia).

Three lux-biosensors were used in this study. The *E. coli* K12 MG1655 (pColD::lux) strain contains the promoter of the colicin D gene, which is part of the SOS regulon; this promoter is activated upon DNA damage by various agents. The MG1655 (pKatG::lux) strain carries the promoter of the catalase gene katG from the oxyRS oxidative-stress regulon, sensitive to hydrogen peroxide (the OxyR sensor protein). The MG1655 (pSoxS::lux) strain contains the promoter of the soxS gene from the soxRS regulon, activated by superoxide anion (the SoxR sensor protein) [42]. The (pKatG::lux) and (pSoxS::lux) strains detect the formation of reactive oxygen species (ROS), which cause oxidative stress and cell damage.

The following positive controls were used:

— For MG1655 pColD::lux and pSoxS::lux: dioxidine (JSC Novosibkhimfarm, Russia) at 0.0005 mmol/mL;
— For MG1655 (pKatG::lux): a 3% hydrogen peroxide solution diluted to 0.03%.

#### 4.6.1. Culture Preparation

Before the lux assay, a liquid culture of each strain was prepared in tubes with 5 mL of LB broth, Miller (VWR International, Radnor, PA, USA): 25 g of dry powder was dissolved in 1 L of water and autoclaved; after autoclaving, 5 µL of ampicillin (final concentration 100 µg/mL) was added to each tube. Bacteria were transferred from a slant (2% LB agar with 100 µg/mL ampicillin) using a 1 µL plastic loop and cultured at 37 °C for 16 h to obtain an overnight culture. In the morning, 2 mL of the overnight culture was transferred to a flask with 20 mL of fresh LB broth and 20 µL of ampicillin and incubated on a shaker at 37 °C (1.5–2 h, 160 rpm) until a turbidity of 3.7 McFarland units (900–1200 × 10^6^ cells/mL) was reached, as measured with a DEN-1 densitometer (Biosan, Riga, Latvia).

#### 4.6.2. Lux Assay Procedure

The experiment was carried out in 96-well white plates. To each well, 20 µL of test substance and 180 µL of bacterial culture were added (substance dilution 1:10). In the co-treatment experiments with extracts and inducers, 160 µL of culture, 20 µL of extract, and 20 µL of inducer or water were added (dilution 1:10). Before luminescence measurement, the cultures were incubated with the test substances at 37 °C. The incubation time was selected individually for each biosensor as the time to reach the maximal luminescence level under the inducer, according to [43]; the optimal incubation times are presented in Table 5. Luminescence was measured on a Stat Fax 4400 (LuMate) plate luminometer (Awareness Technology, Inc., USA), and the results were expressed in RLU.

#### 4.6.3. Bacterial Viability Assay (CFU)

Overnight cultures of MG1655 (pColD::lux) (16 h at 37 °C) were added to fresh LB broth at a 1:10 ratio and incubated for 2 h at 37 °C with aeration (120 rpm). *Rubus saxatilis* extracts or water (control) were then added, and the cultures were incubated for 1.5 h at 37 °C. After incubation, the cultures were serially diluted (10^−1^–10^−6^) and plated on agar plates; colonies grown over 24 h were counted, with dilution factors taken into account, to determine the concentration of viable cells.

#### 4.6.4. Culture Growth Rate Assay

Because *R. saxatilis* extracts have their own intrinsic turbidity, culture growth was assessed from the change in turbidity (ΔE) over time relative to the start of the experiment:

ΔE = E_t − E_0_

where E_t is the turbidity at time t and E_0_ is the turbidity at the start of the experiment. Measurements were taken over 24 h. Dioxidine at 0.0005 mmol/mL served as the reference toxicant in the assay. Variants with and without dioxidine were set up on the same plate. The experiment was performed in three independent replicates.

### 4.7. In Vivo Genotoxic Effects on Drosophila Melanogaster

The genotoxicity of *R. saxatilis* extracts was assessed by single-cell alkaline gel electrophoresis (comet assay) in late second- to early third-instar larvae of the Canton-S line of *D. melanogaster*. Larvae were reared on Alderston medium (250 mL distilled water, 25 g glucose, 25 g yeast, 2 g agar, 500 µL propionic acid) supplemented with the test extracts at the specified concentrations, previously dissolved in distilled water. At least 15–20 larvae were used in each experimental group. Etoposide, a topoisomerase II inhibitor (Sigma-Aldrich, USA), at 0.04% (400 µg/mL) was used as the positive control, added to the medium in the same way as the test extracts. Intact larvae on standard medium without additives served as the negative control. The larvae were homogenised, and the cells were embedded in 0.65% agarose gel and lysed for 1–2 h at 4 °C. Gel electrophoresis was performed at 80 V for 40 min. The preparations were stained with ethidium bromide (50 µg/mL) for at least 15 min, followed by rinsing with distilled water. At least 100 cells per preparation were analysed. The degree of DNA damage was scored on a five-point visual scale: 0—intact DNA, I—minimal damage, II—moderate damage, III—pronounced damage, IV—maximal DNA fragmentation. From these data, the comet assay index (CAI) and the proportion of cells with each damage type were calculated for each experimental group.

### 4.8. Statistical Analysis

Statistical processing and visualisation of the experimental results were performed using GraphPad Prism (version 8.0) and Microsoft Excel 2019. All experiments were carried out in triplicate. Data are presented as arithmetic means ± standard deviation (SD), as indicated in the captions of the corresponding figures.

The normality of the quantitative data was assessed using the Shapiro–Wilk test, and the homogeneity of variances was checked using the Brown–Forsythe and Bartlett tests. When the data met the assumptions of normality and homoscedasticity, the statistical significance of differences between experimental groups and the corresponding control samples (the H_2_O solvent control or the mutagen/dioxidine control) was evaluated using one-way and two-way ANOVA. For subsequent comparisons, Dunnett’s post hoc test was applied for comparisons against a single control and for verifying the concentration-dependent effects of the extracts, whereas Tukey’s post hoc test was used for multiple pairwise comparisons in the Drosophila assays. In datasets where the Brown–Forsythe and Bartlett tests indicated significant heterogeneity of variances, the corresponding post hoc results were interpreted with this limitation in mind.

Half-maximal inhibitory (IC_50_), half-maximal effective (EC_50_), and median lethal concentration (LC_50_) values were calculated by non-linear regression, fitting sigmoidal dose–response curves with a variable slope (Hill slope).

Levels of statistical significance are indicated on the figures as follows: * *p* < 0.05, ** *p* < 0.01, *** *p* < 0.001, **** *p* < 0.0001; non-significant comparisons are denoted “ns”. Differences were considered statistically significant at *p* < 0.05.

### 4.9. Biosafety Statement

Experiments with the genetically modified reporter strains of *Escherichia coli* were performed during a research internship at the Vavilov Institute of General Genetics, Russian Academy of Sciences (Moscow, Russia), in accordance with the applicable requirements and the institution’s internal biosafety rules.

## 5. Conclusions

The present study demonstrated that the extraction solvent and technique are key factors determining the quantitative distribution of phenolic compounds and the biological activity profile of *Rubus saxatilis* leaf and petiole extracts. Comparison of ultrasound-assisted extraction with water versus 50% ethanol, and of ultrasound- versus microwave-assisted extraction at constant solvent, showed that both the solvent and the extraction technique alter the content of flavonoid glycosides and influence the mechanism of antioxidant action. Integrating phytochemical profiling with bacterial lux-biosensors and the in vivo *Drosophila melanogaster* model established that *R. saxatilis* extracts possess pronounced redox-modulatory potential: at the bacterial level they induced neither oxidative stress nor the SOS response, and the decrease in basal stress-response levels together with the attenuation of induced SOS signalling is consistent with a DNA-protective effect at the bacterial level. In the eukaryotic *Drosophila* model, by contrast, the extracts exerted moderate, extraction-method- and concentration-dependent genotoxicity—up to Type III DNA damage for WE, up to Type II for UAE, and only minor (Type I) damage for MAE, all below the etoposide positive control. These divergent, concentration-dependent effects underscore the need for cautious interpretation of the biological activity of polyphenol complexes and the importance of the concentration factor in evaluating their functional action. Overall, these findings extend the understanding of the biological potential of *R. saxatilis* and suggest that extraction method selection meaningfully affects the phenolic profile and biological activity of *R. saxatilis* extracts, providing a methodological basis for future phytopharmaceutical and functional product development, pending further mechanistic and bioavailability studies. Further research should focus on a deeper investigation of the underlying molecular mechanisms, assessment of the bioavailability of the active components, and analysis of long-term exposure effects.

## Figures and Tables

**Table 1:** Identification and content of phenolic compounds in *R. saxatilis* extracts determined by HPLC-UV-ESI-MS.

Peak No.	RT, min	[M−H]^−^ (*m*/*z*)	Compound	WE, mg/g DE	UAE, mg/g DE	MAE, mg/g DE
1	4.79	169	Gallic acid	16.25	17.36	14.68
2	13.95	609	Rutin	ND	7.28	8.92
3	14.70	463	Hyperoside	19.75	6.77	8.70
4	21.77	301	Quercetin	0.11	0.08	0.27

ND—not detected; RT—retention time.

**Table 2:** Radical-scavenging activity (DPPH) of *R. saxatilis* extracts.

№	Extract	IC_50_ (µg/mL) Mean ± SD	Activity
1	MAE	7.44 ± 0.32	Very strong
2	UAE	104.08 ± 12.40	Moderate
3	WE	232.21 ± 5.11	Very weak

by Molyneux, P.

**Table 3:** Ferric reducing antioxidant power (FRAP) of *R. saxatilis* extracts.

№	Extract	EC_50_ (µg/mL) Mean ± SD	Activity
1	WE	52.97 ± 2.80	High
2	MAE	177.49 ± 8.30	Moderate
3	UAE	237.36 ± 11.10	Moderate

**Table 4:** Cytotoxic activity of *R. saxatilis* extracts against *Artemia salina* nauplii.

№	Extract	LC50 (µg/mL)	Meyers Index	Clarkson Index
1	MAE	>1000	Nontoxic	Nontoxic
2	WE	>1000	Nontoxic	Nontoxic
3	UAE	>1000	Nontoxic	Nontoxic

**Table 5:** Characteristics of the *E. coli* MG1655 lux-biosensor strains: specific inducers, working concentrations, optimal incubation times, and induction of luminescence.

Biosensor Strain	Luminescence Inducer	Concentration	Incubation Time, min	Luminescence, RLU ± SD (E_Inducer/E_Water *, Fold)
MG1655 (p*KatG*::*lux*)	H_2_O_2_	0.03%	45	76,872.2 ± 8186 (17.8)
MG1655 (p*SoxS*::*lux*)	Dioxidine	0.0005 mmol/mL	60	25,662.9 ± 1184 (3.2)
MG1655 (p*ColD*::*lux*)	Dioxidine	0.0005 mmol/mL	90	38,576.2 ± 8693 (42.2)

* E—effect.

## Data Availability

The data presented in this study are available on request from the corresponding author.

## Abbreviations

The following abbreviations are used in this manuscript:

**UAE**	ultrasound-assisted extract
**MAE**	microwave-assisted extract
**WE**	water extract
**DPPH**	2,2-diphenyl-1-picrylhydrazyl
**FRAP**	ferric reducing antioxidant power
**HPLC**	high-performance liquid chromatography
**ESI-MS**	electrospray ionization mass spectrometry
**IC_50_**	half-maximal inhibitory concentration
**EC_50_**	half-maximal effective concentration
**LC_50_**	median lethal concentration
**RLU**	relative light units
**CFU**	colony-forming units
**ROS**	reactive oxygen species
**TPTZ**	2,4,6-tris(2-pyridyl)-s-triazine
**SD**	standard deviation
**ND**	not detected
**DE**	dry extract
